# Characteristics of Eye-Position Gain Field Populations Determine Geometry of Visual Space

**DOI:** 10.3389/fnint.2015.00072

**Published:** 2016-01-20

**Authors:** Sidney R. Lehky, Margaret E. Sereno, Anne B. Sereno

**Affiliations:** ^1^Computational Neurobiology Laboratory, The Salk InstituteLa Jolla, CA, USA; ^2^Department of Psychology, University of OregonEugene, OR, USA; ^3^Department of Neurobiology and Anatomy, University of Texas Medical SchoolHouston, TX, USA

**Keywords:** macaque monkey, spatial representation, population coding, active vision, dimensionality reduction, eye movements

## Abstract

We have previously demonstrated differences in eye-position spatial maps for anterior inferotemporal cortex (AIT) in the ventral stream and lateral intraparietal cortex (LIP) in the dorsal stream, based on population decoding of gaze angle modulations of neural visual responses (i.e., eye-position gain fields). Here we explore the basis of such spatial encoding differences through modeling of gain field characteristics. We created a population of model neurons, each having a different eye-position gain field. This population was used to reconstruct eye-position visual space using multidimensional scaling. As gain field shapes have never been well-established experimentally, we examined different functions, including planar, sigmoidal, elliptical, hyperbolic, and mixtures of those functions. All functions successfully recovered positions, indicating weak constraints on allowable gain field shapes. We then used a genetic algorithm to modify the characteristics of model gain field populations until the recovered spatial maps closely matched those derived from monkey neurophysiological data in AIT and LIP. The primary differences found between model AIT and LIP gain fields were that AIT gain fields were more foveally dominated. That is, gain fields in AIT operated on smaller spatial scales and smaller dispersions than in LIP. Thus, we show that the geometry of eye-position visual space depends on the population characteristics of gain fields, and that differences in gain field characteristics for different cortical areas may underlie differences in the representation of space.

## Introduction

Changes in gaze angle can modulate visual responses of neurons in many brain structures and are often summarized by eye-position (EP) gain fields. Gain fields were originally reported in posterior parietal cortex, including areas 7a and lateral intraparietal cortex (LIP; Lynch et al., 1977; Sakata et al., 1980; Andersen and Mountcastle, 1983). Their presence was subsequently found to be widespread, perhaps even ubiquitous, in cortical areas related to both dorsal and ventral visual streams. In addition to confirmations of the presence of gain fields in ***LIP*** or ***area 7a*** (Andersen et al., 1985, 1990b; Squatrito and Maioli, 1996; Siegel et al., 2003; Morris et al., 2013; Sereno et al., 2014), gain fields have also been found in ***V1*** (Weyand and Malpeli, 1993; Guo and Li, 1997; Trotter and Celebrini, 1999; Rosenbluth and Allman, 2002; Durand et al., 2010; Merriam et al., 2013; Strappini et al., 2015), ***V2*** (Rosenbluth and Allman, 2002; Merriam et al., 2013; Strappini et al., 2015), ***V3*** (Galletti and Battaglini, 1989; Merriam et al., 2013), ***V4*** (Bremmer, 2000; DeSouza et al., 2002; Rosenbluth and Allman, 2002), ***V5*** (***MT***; Boynton et al., 1996; Bremmer et al., 1997; DeSouza et al., 2002; Merriam et al., 2013; Morris et al., 2013), ***V6*** (Galletti et al., 1995), **V6A** (Breveglieri et al., 2009), ***MST*** (Squatrito and Maioli, 1996; Bremmer et al., 1997; Morris et al., 2013), ***ventral intraparietal cortex*** (***VIP;*** Duhamel et al., 1997; Morris et al., 2013), ***anterior inferotemporal cortex*** (***AIT;*** Nowicka and Ringo, 2000; Lehky et al., 2008; Sereno et al., 2014), ***frontal eye field*** (***FEF;*** Cassanello and Ferrera, 2007), ***supplementary eye field*** (***SEF;*** Schall, 1991; Schlag et al., 1992), ***dorsolateral prefrontal cortex*** (Takeda and Funahashi, 2002), ***ventrolateral prefrontal cortex*** (Boussaoud et al., 1993), ***dorsal premotor cortex*** (Boussaoud et al., 1998), ***ventral premotor cortex*** (Boussaoud et al., 1993), and the ***hippocampus*** (Nowicka and Ringo, 2000). Eye-position modulations also exist in some non-cortical areas involved in visual processing, namely the ***dorsal lateral geniculate nucleus*** (***LGN;*** Lal and Friedlander, 1989, 1990) and the ***superior colliculus*** (Van Opstal et al., 1995; Stuphorn et al., 2000; Campos et al., 2006).

Eye-position modulations of visual responses are generally believed to play a key role in building spatial representations of the world. The wide range of brain structures showing gain fields indicate that such spatial representations are not merely important for visuomotor control in the dorsal visual stream, as dominates current thinking. Gain fields in the ventral stream are likely to have other purposes, such as providing spatial information about objects and scenes to prefrontal cortex for working memory or cognitive control (e.g., spatial problem solving), as well as spatial encoding of objects and scenes into longer-term memory, necessary for recognition.

Zipser and Andersen (1988) showed through neural modeling that gain fields could be used to compute a coordinate transform from retinotopic space to head-centered space, thereby constructing a head-centered spatial representation of stimulus locations across eye movements. This has remained a common interpretation of the function of gain fields, as has been elaborated in various theoretical studies and reviews (Andersen et al., 1993; Pouget and Sejnowski, 1997; Pouget and Snyder, 2000; Salinas and Thier, 2000; Snyder, 2000; Salinas and Abbott, 2001; Salinas and Sejnowski, 2001; Cassanello and Ferrera, 2007). While eye-position gain fields are sufficient to transform space from retina- to head-centered coordinates, getting to an allocentric spatial representation using the coordinate transform approach would require additional transformations taking various body postures into account (for example, head position through neck proprioceptors, and overall body position through vestibular input).

Pursuing a somewhat different approach, rather than (or in addition to) using gain fields to compute a coordinate transform of the visual field, it is also possible to compute gaze angle directly, by applying population decoding methods to a neural population containing a diversity of gain fields (Bremmer et al., 1998; Boussaoud and Bremmer, 1999; Merriam et al., 2013; Morris et al., 2013; Graf and Andersen, 2014; Sereno et al., 2014). This alternative approach is significant because calculating gaze angle is equivalent to determining the spatial location of a stimulus at fixation in head-centered coordinates. Assuming a fixed body and head position while scanning across the visual field and holding the positions of successively fixated targets in a memory buffer, the relative positions of targets across a scene (i.e., an allocentric representation) can easily be determined (e.g., imagine here a primate sitting in a tree as opposed to a rat rummaging through a garbage strewn alley).

The approach taken in this modeling study to decoding populations of gain fields capitalizes on the information inherent in these relative positions. Specifically, the approach applies multidimensional scaling (MDS), an intrinsic method of population decoding (Lehky et al., 2013), to extract relative eye positions from the responses of gain field populations, keeping stimulus retinal position constant. This modeling builds on previous experimental work using MDS to decode and make a first comparison of spatial encoding derived from populations of gain fields from dorsal and ventral stream neurons (Sereno et al., 2014).

This approach is also conceptually analogous to previous experimental (Sereno and Lehky, 2011) and modeling (Lehky and Sereno, 2011) studies using MDS to extract retinotopic positions (rather than eye positions as is done here) from populations of receptive fields (rather than gain fields), keeping eye position constant. When measuring gain fields the stimulus retinotopic position is held fixed while eye position changes, while when measuring receptive fields eye position is held fixed while stimulus retinotopic position changes.

This approach using MDS differs fundamentally from that taken in a number of previous studies that have used extrinsic decoding methods on gain field populations, such as Bayesian estimation or maximum likelihood estimation (Bremmer et al., 1998; Boussaoud and Bremmer, 1999; Merriam et al., 2013; Morris et al., 2013; Graf and Andersen, 2014). The differences between intrinsic and extrinsic population methods have been discussed in Lehky et al. (2013). For present purposes, perhaps the most noteworthy difference is that intrinsic approaches decode space relationally in a holistic manner, constructing global spatial maps in terms of relative positions, whereas extrinsic methods find absolute positions in an atomistic manner, dealing with each position in isolation from all others.

At present, despite the wide range of brain structures in which gain fields occur, the shapes of gain fields have not been well-defined experimentally, as they have not been measured at high resolution using a large sampling of gaze angles. Although often described as being planar (e.g., Andersen et al., 1993; Bremmer et al., 1998; Boussaoud and Bremmer, 1999), examination of the published data makes it clear that a large majority have more complicated shapes than that. A typical result is the report by Andersen et al. (1985) in which just 39% of gain fields were well fit by a planar regression model. Despite the popularity of planar descriptions, reports of non-monotonic gain fields are commonplace (Sakata et al., 1980; Lal and Friedlander, 1989; Andersen et al., 1990a; Galletti et al., 1995; Squatrito and Maioli, 1996; Rosenbluth and Allman, 2002; Breveglieri et al., 2009) and modeling using non-monotonic gain fields has been performed by Breveglieri et al. (2009). Moreover, the gain fields developed by the Zipser and Andersen (1988) model appear to have a variety of complex shapes that could only be described as planar under a very crude approximation.

Given the lack of precise knowledge about gain field shapes, this modeling study has two major goals. The first goal is to examine how gain field shape affects eye-position spatial representations in general. The strategy will be to compare the recovery of eye positions using several broad categories of gain fields (both monotonic and non-monotonic).

The second goal is to compare model gain field parameters that best explain eye-position spatial maps derived from monkey data (Sereno et al., 2014) obtained from a dorsal stream structure (LIP) and a ventral stream area (AIT). Sereno et al. (2014) found differences between cortical areas in the accuracy with which eye-position visual space could be decoded from populations of gain fields. LIP represented space more accurately than AIT. This difference may relate to functional requirements in the encoding of space needed for guiding visuomotor behaviors vs. object or scene recognition and memory, respectively. Through modeling, we hope to elucidate the key differences in gain fields in dorsal and ventral streams that may prove a crucial first step to better understanding encoding differences in these two major visual processing streams. When examining the LIP and AIT data for goal 2, in addition to MDS we use a genetic algorithm as a data-fitting procedure to help identify what particular gain field characteristics best explain dorsal and ventral spatial maps derived from monkey data.

Given the wide range of brain areas in which gain fields occur, it is likely that spatial representations will differ across those areas, particularly if structures are associated with fundamentally different functions (e.g., dorsal and ventral visual streams). Consequently it seems probable that gain fields in different areas will have different characteristics. Understanding how gain field properties affect spatial representations may provide insight into how space is being used in different cortical regions.

## Methods

Synthetic data for modeling was generated as follows. At a particular eye position, the gain field of a model neuron defines the response to a stimulus. A population of model neurons, each with a different gain field, produces a *response vector* for a given eye position. The number of elements in the response vector is equal to population size. Changing eye position, but keeping the neural population the same, the stimulus the same, and the retinal location of the stimulus the same produces a different response vector, because the response of each gain field depends on eye position. Holding everything constant except eye position ensures that changes in response vectors were due entirely to changes in eye position. Stimulus responses of model neurons were deterministic, without noise.

The set of neural response vectors for different eye positions collectively served as input to a multidimensional scaling (MDS) analysis that recovered eye positions. For example, if population response vectors were determined at 20 eye positions, the MDS analysis would determine the relative locations of those 20 positions in a low dimensional space. Because of the diversity of gain fields within the population, relative responses of neurons in the population change for different eye positions, thus allowing for a population decoding of eye position. This MDS analysis was identical to that which we previously carried out on actual neurophysiological data on gain fields (Sereno et al., 2014), but here we apply it to synthetic data in which the properties of the gain fields could be precisely specified to determine their effect on spatial coding.

### Multidimensional scaling

**Figure 1:**
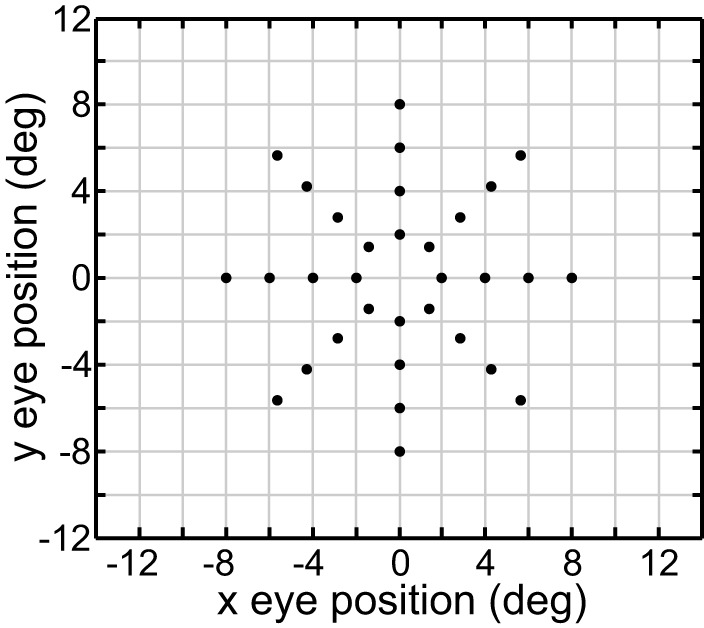
**Set of 40 eye positions used in our examination of the representational capabilities for various kinds of gain fields**.

We performed MDS using the cmdscale command in the Matlab Statistics and Machine Learning Toolbox. For our investigations, we used a standard set of 32 eye positions (EPs). These were arranged in a polar coordinate bull's eye pattern with four concentric circles of EPs, and eight EPs in each circle (Figure 1). Therefore, the data analyzed by MDS was a set of 32 response vectors.

The first step was to calculate the magnitude of the distances or differences between each response vector. All vector differences are placed in a *distance matrix*. For 32 response vectors, all possible differences between them will form a 32 × 32 distance matrix. When calculating the distances between response vectors, a variety of distance measures could be used. We use a correlation measure of distance, *d* = 1 − *r*, where *r* is the correlation coefficient between the elements of two response vectors, as previously described (Lehky and Sereno, 2007).

The distance matrix serves as the input to the MDS algorithm itself (Shepard, 1980; Borg and Groenen, 2010). MDS is a dimensionality reduction algorithm. It takes high-dimensional input and produces a low-dimensional approximation in which values of the distance matrix are kept as close as possible to the original distance matrix.

For example, if there are 500 model neurons in the encoding population, then each eye position is originally represented as a point in a 500-dimensional space. With 32 eye positions, that would be 32 points in a 500-dimensional space. We can't visualize what is being represented in a 500-dimensional space, so MDS reduces the dimensionality subject to the constraint that changes in distance relationships amongst the 32 points are minimized. Because the physical eye positions were in 2D on a flat display, it is particularly useful to use MDS to reduce the neural model's representation of eye position from a high-dimensionality space down to a 2D approximation. That way the physical space and the neural representation of space in the model can be readily compared. For our analysis, MDS, as a dimensionality reduction technique, reduced the original neural representation of 32 eye positions in some high dimensional space (equal to model neuron population size) down to 32 eye positions in a 2D space. That allowed us to determine how accurately the eye positions were being represented in the high-dimensional neural space.

### Procrustes transform

It is important to be able to measure the accuracy of a neural representation. Within extrinsic population coding (Bayesian estimation, etc.; Lehky et al., 2013), accuracy is calculated by comparing the difference between physical and decoded eye positions for individual locations. Within intrinsic population coding such as MDS, accuracy is measured as distortion in the relative positions of multiple points, in a global or holistic fashion. A way to measure accuracy in population decoding in MDS is to use the *Procrustes transform* (Gower and Dijksterhuis, 2004; Borg and Groenen, 2010). The Procrustes transform is a mathematical technique used to indicate how closely the relative positions of two sets of points match each other (in this case, physical eye positions and the neural representations of eye positions). We used the procrustes command in the Matlab Statistics and Machine Learning Toolbox. Since we are interested in relative positions, we don't care if one set of points has been linearly scaled, translated, rotated, or reflected relative to the other, since none of those linear transforms affects relative positions. On the other hand, we are interested if one set of points is a non-linear distortion of the other set (for example, comparing a circle of points with a slightly kidney-shaped set of points). The Procrustes transform is a linear transform that minimizes the difference between two sets of points. After the Procrustes transform finds the best linear fit between two sets of points, then the residual difference between the two sets can be quantified to give a global measure of error between physical eye positions and their neural representation. The global error measure we use is called the *stress* between the two sets of points, defined as:

(1) $$Stress=\sqrt{\frac{\sum_{i}\sum_{j}{\left(d_{ij}-{\hat{d}}_{ij}\right)}^{2}}{\sum_{i}\sum_{j}{\left(d_{ij}-\langle d_{ij}\rangle \right)}^{2}}}$$

In the equation, *d*_*ij*_ is the physical Euclidean distance between eye positions *i* and *j*, ${\hat{d}}_{ij}$ is the distance recovered by MDS from the neural population representation, and 〈·〉 is the mean value operator. Small stress values indicate small error in the representation, or a high degree of accuracy.

### Gain fields

We tested several general categories of gain field shapes: planar, sigmoidal, elliptical, hyperbolic, and complex. Complex gain fields were mixtures of other gain field categories.

#### Planar gain fields

The equation for a planar gain field was:

(2) $$r_{p}=\left(\frac{1}{\sigma }\left(-x\sin\left(\theta \right)+y\cos\left(\theta \right)-\delta_{a}\right)+1\right)∕2$$

**Figure 2:**
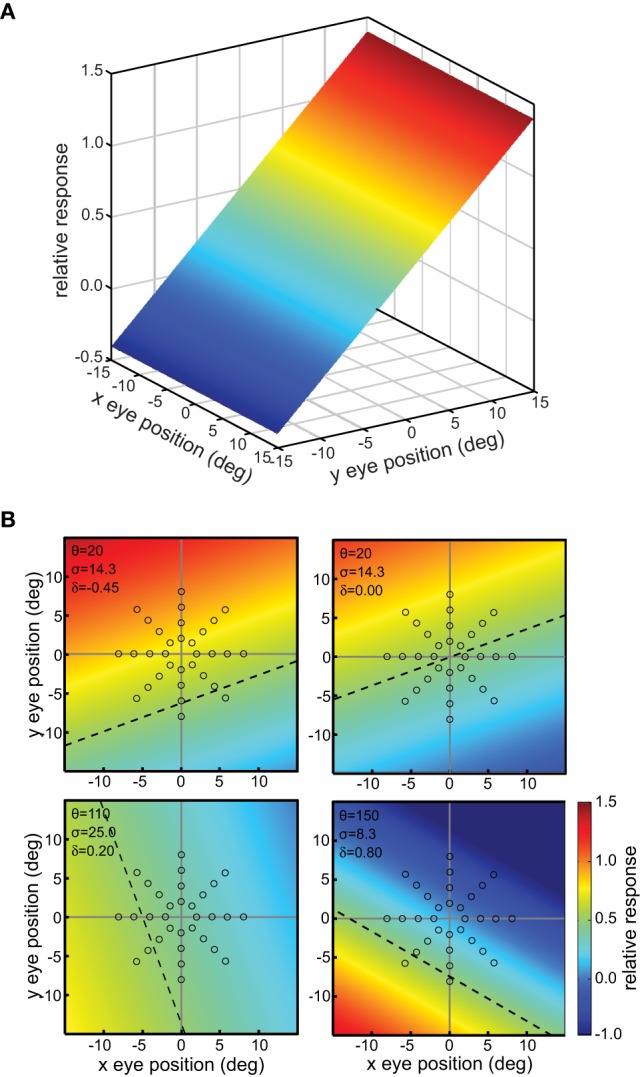
**Example planar gain fields**. **(A)** Three dimensional view. Response is a function of x and y coordinates of eye position, and is indicated by a color code along the z-axis. **(B)** Additional examples of planar gain fields, for different values of three parameters *space constant, orientation*, and *translation*, denoted by σ, θ, and δ, respectively in Equation (3). Relative translations are being used. Here the viewpoint is looking straight down the response axis, with the response level indicated by a color code. The dashed line indicates response values equal to 0.5. The translation parameter δ translates the position where the *r* = 0.5 line occurs. The array of small circles indicates set of eye positions at which the gain field was sampled for computing multidimensional scaling population decoding of eye position, corresponding to the positions shown in Figure 1.

This defined a tilted 3D plane, with *x* and *y* specifying eye position, and the response firing rate *r*_*p*_ forming the third (color-coded) dimension (Figure 2A).

The space constant of the gain field was given by σ, the reciprocal slope of the plane. It is a measure of how rapidly responses change within the gain field. We parameterize planar gain fields in terms of space constant rather than slope in order to keep terminology and mathematical notation the same as with other classes of gain fields, such as elliptical paraboloids, which cannot be simply parameterized in terms of a slope parameter. Gain field orientation was given by θ in degrees. Orientation was defined by the gain field axis within the x-y plane showing the slowest rate of change. For planar gain fields, orientation was the orientation of the iso-response contours. The parameters σ and θ are equivalent to specifying the elevation and azimuth of the gain field in spherical coordinates. The third parameter δ_*a*_ was the absolute translation of the gain field in degrees, in the direction orthogonal to the orientation θ (the subscript *a* indicates absolute translation). Translation defines the position where the midpoint of the firing range occurs, which is 0.5, with firing range defined over the population rather than individual neurons. When δ_*a*_ is zero, the gain field response has a value of 0.5 at central fixation (eye position *x* = 0, *y* = 0). In Equation (2), adding 1 and dividing by 2 sets the value of 0.5 at central fixation for the untranslated gain field. Several example planar gain fields with various parameter values are shown in Figure 2B.

A variant of the planar gain field with relative translation rather than absolute translation was specified by the following equation:

(3) $$r_{p}=\left(\frac{1}{\sigma }\left(-x\sin\left(\theta \right)+y\cos\left(\theta \right)\right)-\delta_{r}+1\right)∕2$$

Here the translation parameter δ_*r*_ (with subscript *r* to specify relative) was not specified in degrees, but rather relative to the value of the space constant parameter σ. For example, δ_*r*_ = 0.5 means that the translation was 0.5 times the value of σ. Thus, this enforced a correlation between the space constant of the gain field and its translation: within a population, gain fields with small space constants had small translations while gain fields with large space constants had large translations. We found for planar gain fields that the version with relative (correlated) translations (Equation 3) was able to reconstruct eye-position visual space slightly more accurately than absolute translations (Equation 2).

We created a diverse population of 10,000 gain fields by generating random values for the parameters. The space constant parameter σ was uniformly distributed over the range 4–40 (i.e., slope values of 0.25–0.025). The orientation parameter θ was uniformly distributed on a linear scale over the range 0–360. The translation parameter δ_*r*_ set in relative terms, was uniformly distributed on a linear scale over the range −1.0 to 1.0 using Equation (3).

#### Sigmoidal gain fields

**Figure 3:**
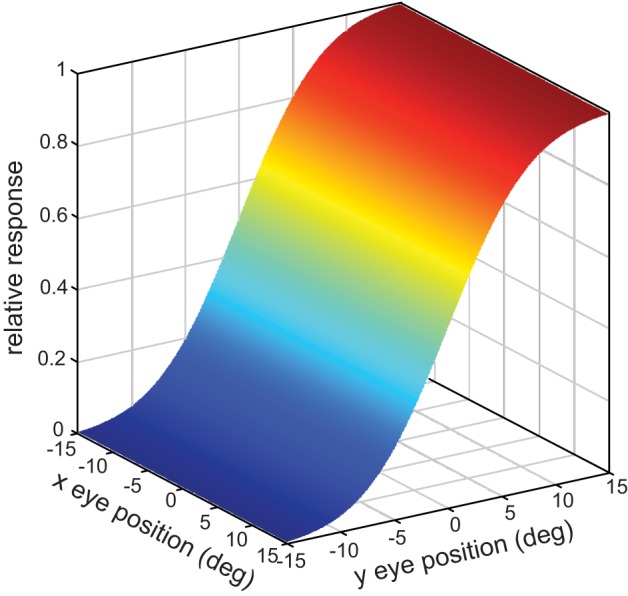
**Example of a sigmoidal gain field, in three-dimensional view**.

Sigmoidal gain fields had a sigmoidal cross section, but were otherwise similar to planar gain fields (compare Figures 2A, 3). The equation for a sigmoidal gain field was:

(4) $$r_{s}=\left(\text{erf}\left[\frac{1}{\sigma }\left(-x\sin\left(\theta \right)+y\cos\left(\theta \right)-\delta_{a}\right)\right]+1\right)∕2$$

where translation δ_*a*_ was in absolute terms (in degrees). This is the same as that for a planar one, except for the addition of an error function erf transform (which is the integral of a Gaussian function) to provide the sigmoidal shape. When translation was in relative terms (relative to σ), the equation was:

(5) $$r_{s}=\left(\text{erf}\left[\frac{1}{\sigma }\left(-x\sin\left(\theta \right)+y\cos\left(\theta \right)\right)\right]-\delta_{r}+1\right)∕2$$

Parameter values for the sigmoidal gain field population were randomly distributed in the same manner described above for planar gain fields, with the population size again being 10,000.

#### Elliptical paraboloid gain fields

Gain fields with an elliptical paraboloid shape were given by:

(6) $$\begin{array}{l} r_{e}=1-\text{erf}[{\left(\frac{1}{\sigma }\left(x\cos\left(\theta \right)+y\sin\left(\theta \right)-\cos\left(\theta -\phi \right)\delta_{a}\right)\right)}^{2}\\ \;+\;\rho {\left(\frac{1}{\sigma }\left(-x\sin\left(\theta \right)+y\cos\left(\theta \right)+\sin\left(\theta -\phi \right)\delta_{a}\right)\right)}^{2}] \end{array}$$

with absolute translations δ_*a*_ and:

(7) $$\begin{array}{l} r_{e}=1-\text{erf}[{\left(\frac{1}{\sigma }\left(x\cos\left(\theta \right)+y\sin\left(\theta \right)\right)-\cos\left(\theta -\phi \right)\delta_{r}\right)}^{2}\\ \;+\;\rho {\left(\frac{1}{\sigma }\left(-x\sin\left(\theta \right)+y\cos\left(\theta \right)\right)+\sin\left(\theta -\phi \right)\delta_{r}\right)}^{2}] \end{array}$$

with relative translations δ_*r*_. These equations have the general elliptical form *r* = *Ax*^2^ + *By*^2^, elaborated to include rotations and translations. The elliptical paraboloids were defined to be convex (peaks) rather than concave (valleys).

Elliptical paraboloid gain fields included the same parameters σ, θ, and δ as the planar and sigmoidal gain fields. They also had two additional parameters. The first was translation direction ϕ in degrees. (Planar and sigmoidal gain fields can't have a variable translation direction because one axis is constant and a translation component along that axis causes no change, forcing the translation direction to always be orthogonal to the constant axis.) The second additional parameter was the ratio ρ of the major axis to minor axis.

For elliptical paraboloid gain fields, the space constant σ was set with a uniform random distribution over the range 20–60, and orientation θ (orientation of the major axis) was set uniformly over the range 0–360. Translation was defined in absolute terms (Equation 6), with δ_*a*_ uniformly distributed over the range −15 to 15. Using relative translation (Equation 7) rather than absolute translation gave better results for elliptical paraboloid gain fields. Translation direction ϕ was set orthogonal to the orientation of the major axis (i.e., ϕ = θ + 90). The axis ratio parameter ρ was uniformly distributed over the range 1–5.

**Figure 4:**
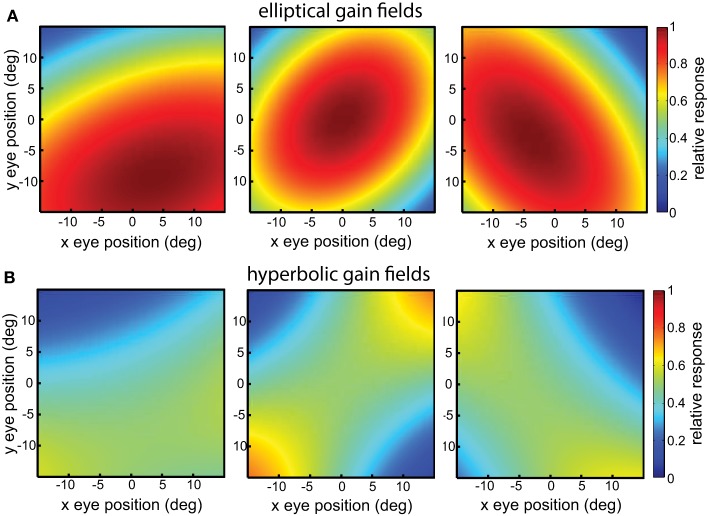
**Example gain fields**. **(A)** Elliptical paraboloid gain fields. **(B)** Hyperbolic gain fields.

Elliptical paraboloids were transformed by a sigmoidal error function in order that its values were bounded in the range [0 1], which distorts the shape slightly from a pure paraboloid. Figure 4A shows example elliptical paraboloid gain fields.

#### Hyperbolic paraboloid gain fields

Hyperbolic paraboloid gain fields were given by:

(8) $$\begin{array}{l} \;r_{h}=(\text{erf}[{\left(\frac{1}{\sigma }\left(x\cos\left(\theta \right)+y\sin\left(\theta \right)-\cos\left(\theta -\phi \right)\delta_{a}\right)\right)}^{2}\\ -\;\rho {\left(\frac{1}{\sigma }\left(-x\sin\left(\theta \right)+y\cos\left(\theta \right)+\sin\left(\theta -\phi \right)\delta_{a}\right)\right)}^{2}]+1)∕2 \end{array}$$

with absolute translations δ_*a*_ and:

(9) $$\begin{array}{l} \;r_{h}=(\text{erf}[{\left(\frac{1}{\sigma }\left(x\cos\left(\theta \right)+y\sin\left(\theta \right)\right)-\cos\left(\theta -\phi \right)\delta_{r}\right)}^{2}\\ -\;\rho {\left(\frac{1}{\sigma }\left(-x\sin\left(\theta \right)+y\cos\left(\theta \right)\right)+\sin\left(\theta -\phi \right)\delta_{r}\right)}^{2}]+1)/2 \end{array}$$

with relative translations δ_*r*_. These equations have the general hyperbolic form *r* = *Ax*^2^ − *By*^2^, again elaborated to include rotations and translations. The parameters for hyperbolic paraboloids have the same meanings as for elliptical paraboloids, and the hyperbolic population had the same range of parameter values as the elliptical one. Figure 4B shows example hyperbolic paraboloid gain fields.

#### Complex gain fields

We created complex gain fields by combining sigmoidal (*r*_*s*_), elliptical (*r*_*e*_), and hyperbolic (*r*_*h*_) gain fields:

(10) $$r_{c}=\frac{1}{3}\left(r_{s}+r_{e}+r_{h}\right)$$

**Figure 5:**
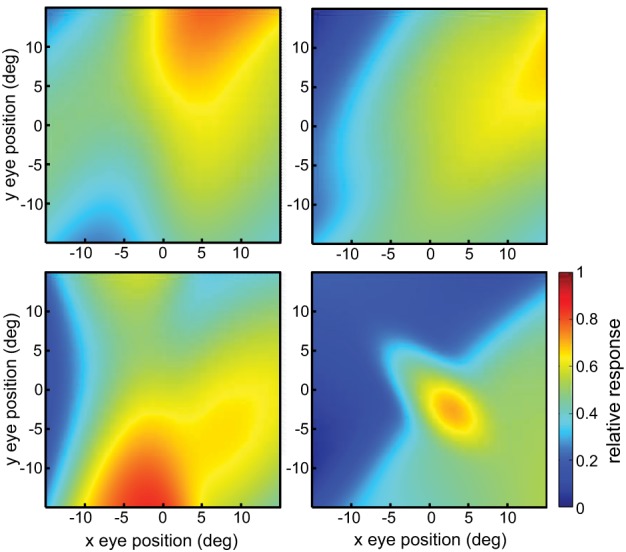
**Example complex gain fields**.

Combining three gain field categories in this manner means that each complex gain field had 13 parameters. For all three components, the space constant parameter σ was uniformly distributed over the range 4–60, and the orientation parameter θ was uniformly distributed over the range 0–360. We used the absolute translation parameter δ_*a*_ for all three components, which was uniformly distributed over the range −15 to 15. For the additional two parameters in the elliptical and hyperbolic components, the translation direction parameter ϕ was orthogonal to the orientation of the major axis (ϕ = θ + 90), and the axis ratio parameter ρ had a uniform random distribution over the range 1–5. Figure 5 shows example complex gain fields.

### Using a genetic algorithm to create specified spatial maps

We have shown that eye-position spatial maps differ depending on whether they were derived from data in LIP or AIT (Sereno et al., 2014; **Figures 13A,B**). Briefly, those experimental results involved a task that required the monkeys to fixate the same stimulus at different eye positions while neurons were recorded using microelectrodes. That allowed population response vectors to be determined for different eye positions. The response vectors were analyzed using MDS in a manner identical to that described here, producing eye-position spatial maps for the two brain areas.

We wished to quantify the differences between AIT and LIP gain fields underlying those different spatial maps. Whereas before we were using MDS and the Procrustes transform to generate a spatial map from a population of gain fields, here we invert the process and use a genetic algorithm to create a population of model gain fields from a spatial map. We used a genetic algorithm to create two populations of model complex gain fields that reproduced the characteristics of LIP or AIT spatial maps, and then examined the manner in which gain field parameters differed for those two populations.

Each complex gain field in the model population was defined by 13 parameters (three for the sigmoidal component, five for the elliptical component, and five for the hyperbolic component). Therefore, for a large population there were too many parameters to set by hand, and we turned to using a genetic algorithm (Mitchell, 1996) to automate the fitting process. The genetic algorithm was used purely as a mathematical method for producing model gain field populations that corresponded to specified eye-position spatial maps, and was not intended to mimic a biological process or the development of gain field populations.

**Figure 6:**
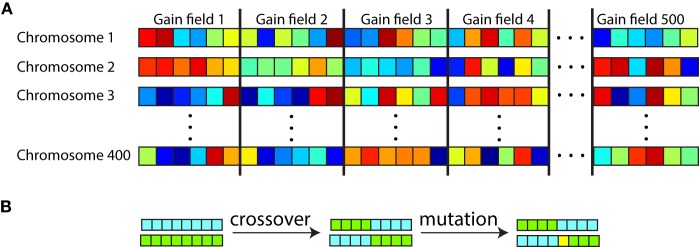
**Organization of chromosomes within the genetic algorithm used to generate populations of gain fields that produce eye-position spatial maps with particular desired characteristics**. **(A)** Each chromosome consisted of a string of parameter values defining gain fields, with parameters for all gain fields in the population concatenated. Each square represents one parameter value, with six parameter values per gain field in this diagram. Different colors represent different numerical values for the parameters. There were 500 gain fields represented on each chromosome (population), and 200–400 chromosomes depending on the run. **(B)** Novel gain field populations were generated through crossover between existing chromosomes and through mutation. Chromosomes with high fitness (those corresponding to gain field populations that generated spatial maps with small errors from the target spatial map) were preferentially reproduced for the next generation. The genetic algorithm was used as a mathematical formalism for creating eye-position spatial maps with particular characteristics, and was not intended to literally represent a biological process.

We used the genetic algorithm software included in the Matlab Global Optimization Toolbox. All the parameters for all the gain fields in a population were listed in a single long sequence to form a chromosome (Figure 6A). With 13 parameters per gain field, and 100 gain fields in the population during preliminary studies, that led to a string of 1300 parameters in a chromosome. There were 200 chromosomes in the genetic population for the preliminary studies, with each chromosome carrying a complete representation of a gain field population. The gain field parameters within each chromosome were initialized to random values. Therefore, with 200 chromosomes there were 200 different random populations of gain fields attempting to produce the specified spatial map.

Starting from this initialization state, each generation of the genetic algorithm involved a two-step process. The first step evaluated the fitness of each chromosome (each string of parameters defining a gain field population) by how well the spatial map produced by the population matched the target spatial map. The second step selectively reproduced the chromosomes with highest fitness (smallest error), and then performed mutations and crossovers on the new chromosome set to introduce novelty (Figure 6B). Going to the next generation, the new set of chromosomes was again evaluated for fitness, and so forth. The loop terminated when the spatial map generated by the best chromosome matched the target spatial map within a specified error tolerance. If the genetic algorithm failed to reach that tolerance within 600 generations the run was aborted and a new run started.

To evaluate the fitness of each chromosome, all the gain field parameters contained within the chromosome were decoded to produce a gain field population. This gain field population was then used to create a spatial map, using multidimensional scaling and the Procrustes transform in the manner described above. Then an error measure between this computed spatial map and the target spatial map was calculated as the vector norm of all the distances between corresponding points in the two maps. This Euclidean error measure indicated the fitness of the chromosome, with small error corresponding to high fitness.

Various other parameters governing operation of the genetic algorithm were set to the default values for the software, as described in Matlab documentation or as listed by the command gaoptimset (@ga) if the Global Optimization Toolbox is installed. Some of the more important default parameters used were a mutation rate of 0.01, a crossover rate of 0.80, and selection of chromosomes within the top 5% of fitness for reproduction to the next generation.

For initial modeling with the genetic algorithm we used idealized versions of the eye-position spatial maps from the monkey data, with a different target map used for modeling LIP and AIT. In later runs, the actual spatial maps from the data were modeled. The idealized LIP target map was spatially veridical, consisting of a polar grid as shown in Figure 1. There were four rings of points at eccentricity radius *r* = [2 4 6 8] degrees. The idealized AIT target map was spatially distorted through the transformation *r*′ = 0.143*r*^1.8^. This transform compressed the spatial map toward the center (**Figure 13D** illustrates what this compressed spatial map looks like). Given that the actual LIP and AIT spatial maps derived from data were based on a small and noisy data sample, we created these LIP and AIT target maps as idealizations of what we viewed as the essential characteristics of the LIP and AIT maps.

Following preliminary studies using the genetic algorithm, we found that only six of the 13 parameters defining a complex gain field were important in explaining the difference between LIP and AIT spatial maps. These were the space constant σ and translation δ parameters for each of the three components included in a complex gain field (sigmoidal, elliptical, and hyperbolic components). The other seven parameters (orientation θ for all three components, as well as translation direction ϕ and axis ratio ρ for elliptical and hyperbolic components), after application of the genetic algorithm, were found to follow an approximately uniform distribution that did not differ in a notable manner between AIT and LIP. We therefore decided to simplify the model by holding those parameters fixed with uniform distributions specified in advance rather than having them trained by the algorithm. This led to a new set of genetic algorithm runs using only six parameters per gain field rather than thirteen, allowing them to run more quickly.

For this second set of runs the gain field population size was increased to 500, and the number of chromosomes within each generation was increased to 300. Results from the second set of runs are reported below. The gain field population size of 500 was determined by computer speed, allowing a run to finish in a reasonable amount of time. Results from multiple 500-unit populations were pooled when examining the statistical distributions of parameter values that had been created by the genetic algorithm.

In addition to using a genetic algorithm to create a model gain field population that generated idealized spatial maps for AIT and LIP, we also used the algorithm to create a population that could generate the actual AIT and LIP spatial maps from the monkey data. Because the actual spatial maps showed orientation anisotropy (i.e., were not circularly symmetric), perhaps due to noise in a small data sample or the fact that recordings were made from a single hemisphere, we included the orientation parameter θ for all three complex gain field components amongst the parameters set by the genetic algorithm. That increased the number of parameters per complex gain field from six, for idealized spatial maps, to nine for actual spatial maps. Because actual spatial maps were more irregular than idealized spatial maps, they were more difficult for the genetic algorithm to learn. We therefore increased the number of chromosomes in the algorithm from 300 to 400 to provide a richer sampling of the parameter space when working with actual spatial maps.

## Results

### Accurate representation of space with a broad diversity of shapes in the gain field population

**Figure 7:**
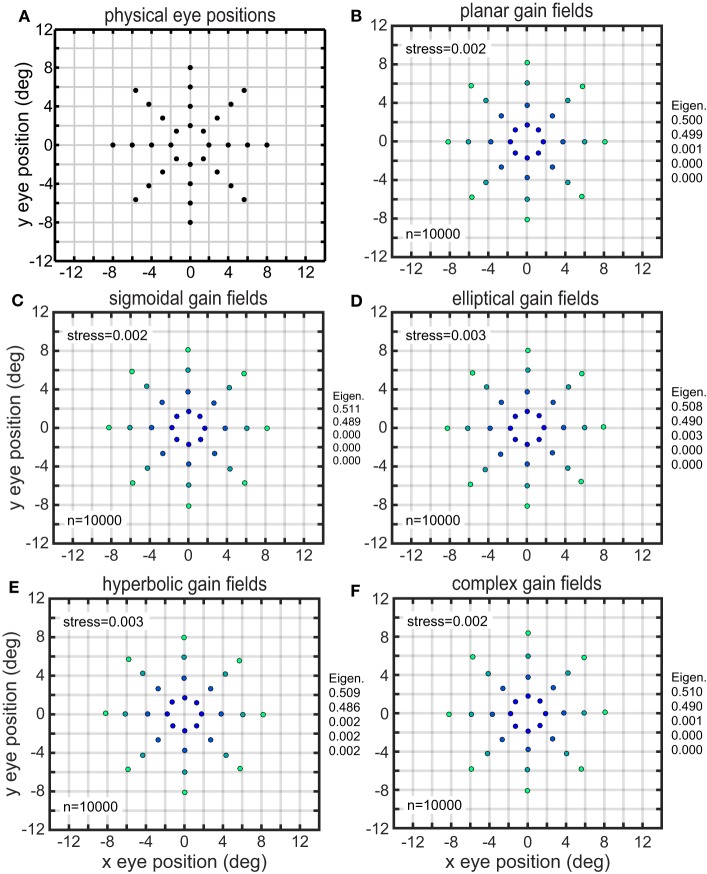
**Recovery of eye positions for different categories of gain fields**. **(A)** Physical eye positions. **(B)** Planar gain fields. **(C)** Sigmoidal gain fields. **(D)** Elliptical gain fields. **(E)** Hyperbolic gain fields. **(F)** Complex gain fields. In all cases accuracy is excellent (low stress), indicating weak constraints on what shapes gain fields may assume and still perform successfully. Population size is indicated by *n*. Normalized eigenvalues of the MDS transform are displayed. Points are color coded according to eccentricity to aid visualization.

The results of the MDS analysis for all five gain field categories are shown in Figure 7. The figure contains two measures of accuracy. The first is stress as defined in Equation (1), with smaller stress indicating a more accurate representation. By convention, stress values below 0.1 are considered indicative of a satisfactory level of accuracy. The second is normalized eigenvalues of the MDS transform (normalized to sum to 1.0), which indicate the fraction of variance in the data accounted for by each MDS dimension. Ideally the normalized eigenvalues should be 0.5 for the first two dimensions and zero for all other dimensions. The points in Figure 7 are color coded for eccentricity. In this figure, which shows accurate representations of space, the color coding is not necessary, but for highly distorted and folded representations as occur in some of the subsequent figures the color coding helps to better identify points.

Both measures of accuracy, stress and eigenvalues, indicate that all categories of gain fields, namely planar, sigmoidal, elliptical paraboloid, hyperbolic paraboloid, and complex, were capable of accurate representations of eye positions. The accuracy of the spatial representations is clear from simple visual inspection of the figure, comparing physical eye positions (Figure 7A) with recovered eye positions in all other panels, as well as from stress values and eigenvalues. Accurate representations occurred when a population of model neurons contained a broad diversity of gain fields, the result of having gain field parameters distributed over a wide range of values across the population. We shall see below that results deteriorate if gain field parameters in the population were restricted to falling within a narrow range.

Results varied slightly depending on the details of how the random parameters were set for the gain field population, but these were small effects not of great importance. Some of these effects are described below.

Parameters had a uniform random distribution over some specified interval. The uniform distribution was generally generated with the parameter expressed on a linear scale. However, for the space constant σ, having that parameter expressed either on a linear or logarithmic scale when generating the uniform distribution both seemed as reasonable biological possibilities and we examined both, finding small differences in the resulting accuracy of the spatial maps. For planar gain fields, recovery of eye-position space was slightly more accurate using a logarithmic scale (stress = 0.002, as shown in Figure 7B) compared to a linear scale (stress = 0.011). Sigmoidal gain fields produced similar results. These changes are small enough not to be noticeable when comparing plots of recovered eye position, and both stress values are small relative to the convention that stress values below 0.1 reflect a good reconstruction of the underlying physical relationships. This difference between linear and logarithmic scales almost entirely disappeared when planar or sigmoidal components were embedded within complex gain fields. In contrast to planar and sigmoidal gain fields, for elliptical and hyperbolic gain fields it made no difference (to three significant figures) whether the scale parameter was uniformly distributed on a linear or logarithmic scale.

Another variation in setting parameter values was to change whether the translation magnitude δ was given in absolute terms (in degrees; Equation 2), or if it was given relative to (and perfectly correlated with) the value of the space constant parameter σ (Equation 3). For planar gain fields, specifying relative offsets produced more accurate results (stress = 0.002) than absolute offsets (stress = 0.015), again with similar results for sigmoidal gain fields. For elliptical and hyperbolic gain fields, the opposite held true. Absolute translations produced better decoding results (stress = 0.003) than relative translations (stress = 0.019). Again, these differences were quite minor. All these effects were substantially diminished for components embedded in complex gain fields.

A third variation in setting parameter values was to change whether translation direction ϕ was set with a uniform random distribution, or if it was set orthogonal to the major axis of elliptical and hyperbolic gain fields (that is, perfectly correlated with the orientation parameter θ, such that ϕ = θ + 90). (The translation direction parameter does not exist for planar and sigmoidal gain fields, as explained in the Methods Section). Reconstruction of visual space was better when orthogonal translation directions were used. With orthogonal translation directions, stress = 0.003 for both elliptical and hyperbolic paraboloid gain fields (Figures 7D,E). Using uniform random translation directions, stress increased to 0.008 for elliptic paraboloid gain fields, and 0.015 for hyperbolic paraboloid gain fields. These relatively inconsequential effects remained when elliptical and hyperbolic components were embedded in complex gain fields.

### Variability in recovered eye positions

Although firing rates for these gain field populations were noise-free, there was still stochastic uncertainty in the recovery of eye positions because the parameters describing gain field characteristics were randomly set. Different random populations of gain fields led to slightly different estimates of eye positions.

**Figure 8:**
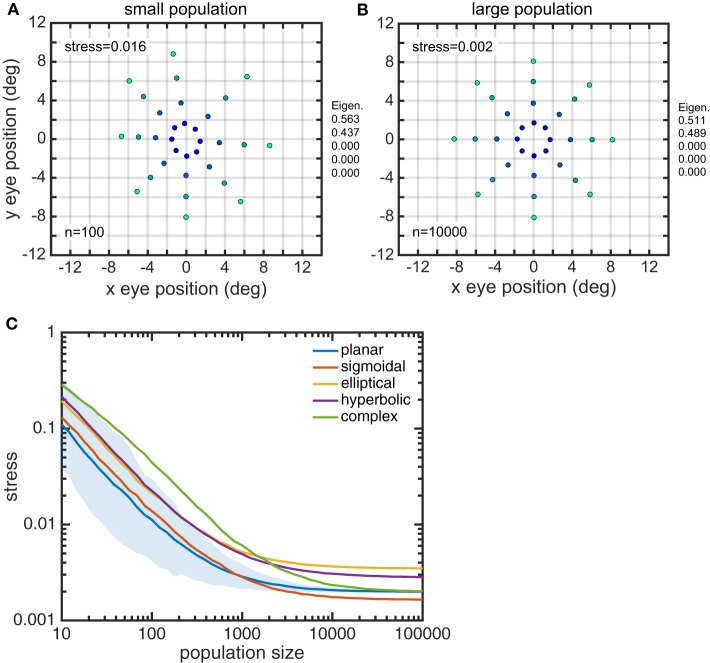
**Effect of gain field population size on accuracy of recovered eye positions, as measured by MDS stress**. **(A)** Recovered eye positions for small population, size *n* = 100. **(B)** Recovered eye positions for large population, size *n* = 10000. **(C)** MDS stress, as a function of gain field population size for different classes of gain fields. Each curve is the average of 1000 replications. Standard deviation shown for planar gain fields.

**Figure 9:**
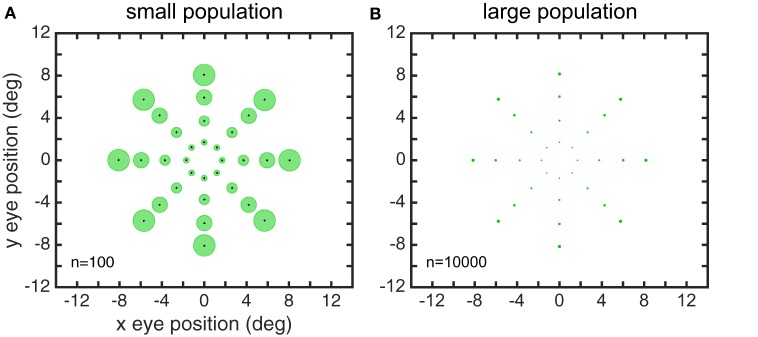
**Precision of recovered eye positions, as measured by circular error probability (CEP)**. CEP indicates the radius within which 50% of estimated eye positions will fall for different random gain field populations. **(A)** CEP for small population, *n* = 100. **(B)** CEP for large population, *n* = 10.000. Both these examples used sigmoidal gain fields.

Larger gain field populations produced more accurate estimates of eye position. The global error in reconstructing eye-position spatial maps, measured by stress, decreased with gain field population size (Figure 8). For large population sizes (≥10, 000) differences in stress for different gain field categories were statistically significant (for this noise-free system). However, stress for all categories were all so small (range: 0.0016–0.0035) that the differences were probably not functionally significant, and any gain field category could plausibly be used biologically. Larger population sizes also produced more precise estimates of eye position, as measured locally for each eye position by the circular error probability (CEP; Figure 9). CEP indicates the radius within which 50% of eye position estimates will fall.

These results show the variability of eye-position decoding for different random samplings of gain field populations. Accurate and precise recovery could be achieved with substantially smaller populations that were carefully selected rather than random. This could occur, for example, by using plastic changes in gain fields properties coupled with an error correction process, in order to homogenously span the gain field parameter space when recovering spatial maps.

### Distorted representations of space when gain field population diversity was reduced

When the diversity of gain fields in a population was reduced by restricting the range of parameter values, the representation of eye-position space in most cases became heavily distorted. The nature of the spatial distortion depended on which parameter was restricted, and also could depend on the general category of the gain fields included in the population (planar, elliptical, etc.).

**Figure 10:**
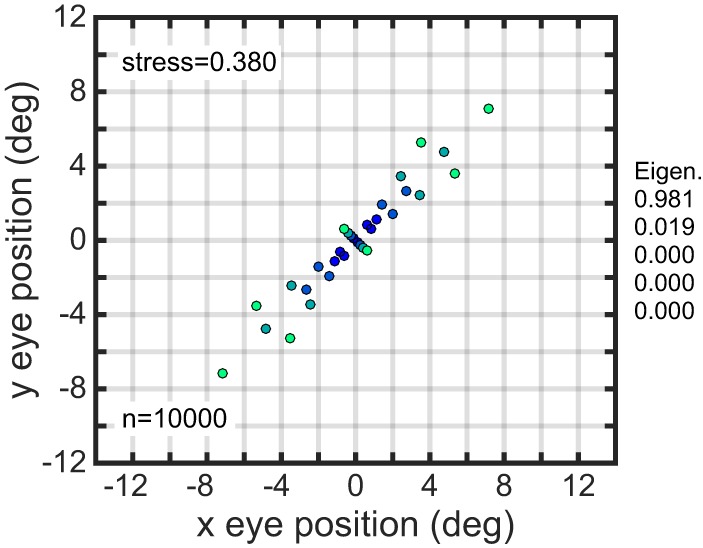
**Distorted recovery of eye positions when the range of orientations θ within the gain field population was restricted**. This example was from a population of planar gain fields, but all categories of gain fields behaved similarly. Physical eye positions were as shown in Figure 1.

If gain field orientations θ in the population were restricted to a small range of values, the resulting eye-position space showed orientation specific distortions (Figure 10, using planar gain fields). While the usual populations (such as those illustrated in the different panels of Figure 7) had gain field orientations covering a full 360°, the population underlying Figure 10 had its orientations restricted to a range one tenth that value, or 36°. The mean orientation in this restricted range for was −45°. Therefore, the contracted portion of the eye-position space was orthogonal to the missing orientations in the population (under our convention that defines 0° orientation as the direction of the iso-response contours in a plane or the direction of the major axis in elliptical and hyperbolic paraboloids). Essentially the same result was observed for all gain field categories, and not just for planar ones as used in this illustration.

**Figure 11:**
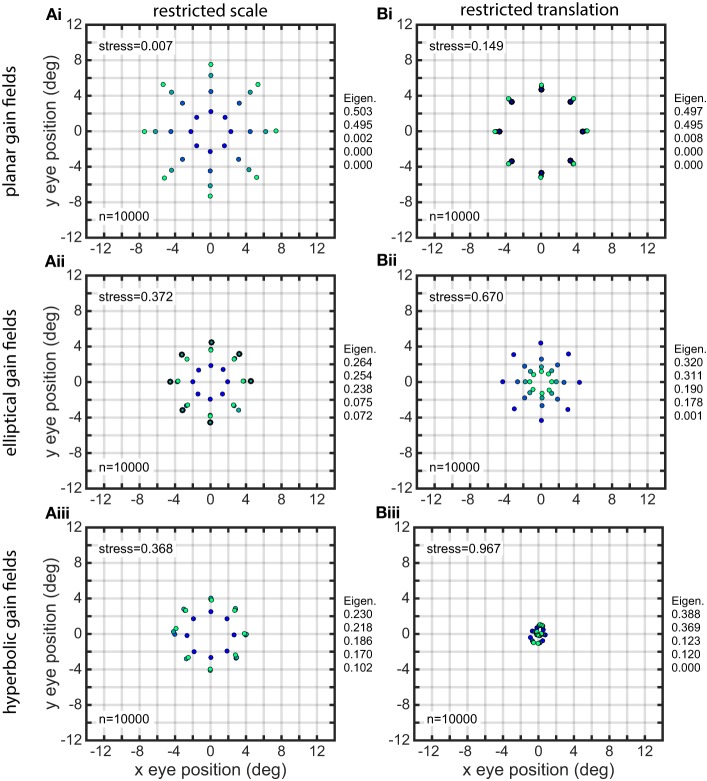
**Distorted recovery of eye positions when the ranges of spatial scale or translation within the gain field population were restricted**. **(A)** Recovery of eye positions when the space constant parameter σ was restricted to a narrow range of small values across the gain field population. **(i)**. Planar gain fields. **(ii)**. elliptical paraboloid gain fields. **(iii)**. Hyperbolic paraboloid gain fields. σ = 4 − 8 in all cases. **(B)** Recovery of eye positions when the translation parameter δ was restricted to a narrow range of small values across the gain field population, centered on zero translation. This is equivalent to saying the gain field populations had small *dispersions*. **(i)**. Planar gain fields (δ_*r*_ = −0.1 to 0.1). **(ii)**. elliptical paraboloid gain fields (δ_*a*_ = −1.5 to 1.5). **(iii)**. Hyperbolic paraboloid gain fields (δ_*a*_ = −1.5 to 1.5). Eye-position visual space is severely disrupted for gain field populations with small dispersions. Physical eye positions were as shown in Figure 1.

The effects of restricting the space constant parameter σ to small values (4.0–8.0) are shown in Figure 11A, for three classes of gain fields. (Sigmoidal gain fields are not included because they don't differ significantly from planar ones). The spatial representation produced by planar gain fields was modestly degraded under this condition, while the spatial representations produced by elliptical and hyperbolic gain fields were severely disrupted. Note that the spatial representations for elliptical and hyperbolic gain fields are so severely distorted that they no longer preserve topological relationships of the underlying physical positions. The positions in the outermost ring (lightest green) have contracted to such a great extent that they are now located inside some of the inner rings, as is apparent from inspection of the coloring of the dots representing eye-positions. In contrast to the severe degradation that occurs when space constant values σ in the population were restricted to a narrow range of small values, there were minor effects in all gain field categories when σ was restricted to a narrow range of large values.

The effects of restricting the translation magnitude parameter δ to a narrow range of small values are shown in Figure 11B. This manipulation caused spatial representations to become severely disrupted primarily with respect to eccentricity. Restricting gain fields to small translation values is equivalent to saying the gain field population had a small *dispersion* [using terminology introduced in Lehky and Sereno (2011)]. The distorted spatial maps for planar gain fields are shown in Figure 11Bi, where relative translation δ_*r*_ was uniformly distributed over the narrow range −0.1 to 0.1 (small dispersion), compared to accurate decoding in Figure 7B where δ_*r*_ was over the broad range −1.0 to 1.0 (large dispersion). Similar results occurred if there was a restricted range of small absolute translations rather than small relative values. Distorted spatial maps for elliptical and hyperbolic gain fields are shown in Figures 11Bii,iii, using absolute translations δ_*a*_ uniformly distributed over a narrow range of small values −1.5 to 1.5 (small dispersion). These distorted maps can be compared to the accurate decoding shown in Figures 7D,E, where δ_*a*_ extended over a broad range of values −15 to 15 (large dispersion).

If translation δ was set to zero for the entire population rather than a narrow range of small values, then the spatial map for planar and sigmoidal gain fields collapsed to a single ring, with points at all eccentricities exactly superimposed. The spatial maps for elliptical and hyperbolic paraboloids collapse to a single location at the center. When translation was set to zero for the entire population, this means the population had zero dispersion, and the resulting severe disruption to spatial maps emphasizes the importance of population dispersion in decoding space.

When translation values were restricted to a narrow range of large values rather than a narrow range of small values, spatial decoding for planar gain fields was still severely distorted, looking essentially the same as shown in Figure 11Bi. The results were dramatically different for elliptical and hyperbolic gain fields, for which recovered spatial maps showed little distortion and looked similar to those in Figure 7.

Restricting the translation direction parameter ϕ to a narrow range of values in elliptical and hyperbolic gain fields caused strong orientation-specific distortions in the recovered spatial map. The results were very similar to Figure 10, which was produced when the orientation parameter θ was restricted. Finally, restricting the parameter ρ, the ratio of the major axis to minor axis in elliptical and hyperbolic gain fields, to a narrow range of values had virtually no effect. The recovered visual space remained highly accurate.

Complex gain fields were less sensitive to restricting parameter values than their three gain field components. If restricting a parameter, for example orientation θ, caused severe distortion for sigmoidal, elliptical, and hyperbolic gain fields when each was examined individually, combining those three components into a complex gain field led to a lower level of distortion.

**Figure 12:**
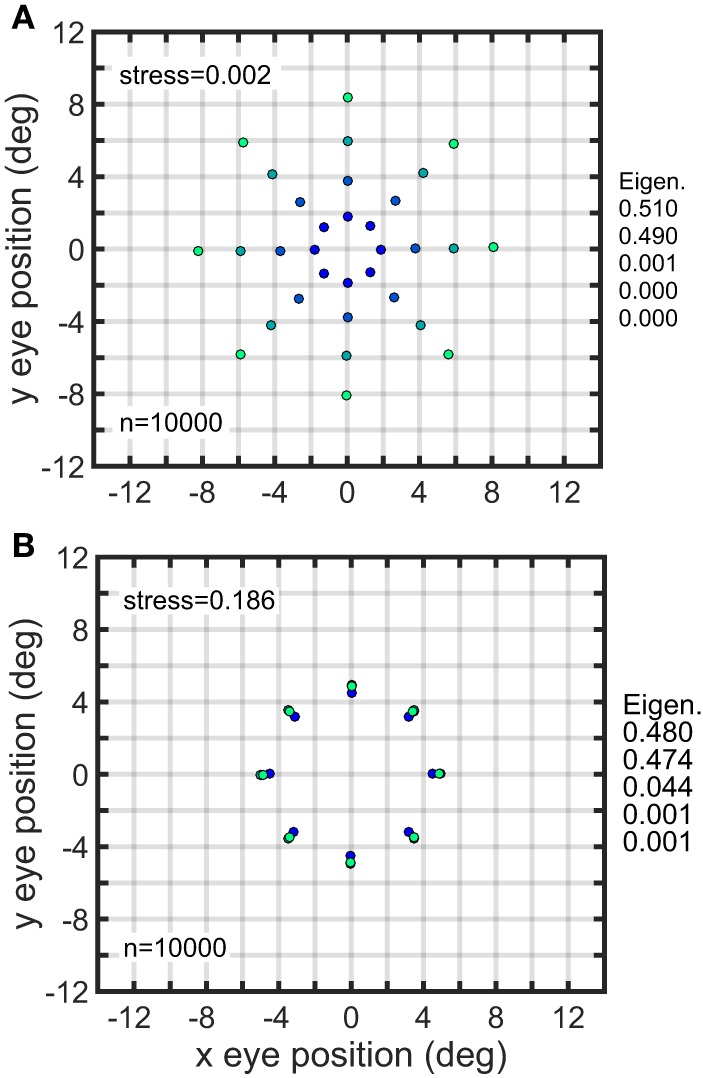
**Effect of normalizing all gain fields in a population to have same mean value. (A)** Recovery of eye positions from a population of unnormalized complex gain fields. **(B)** Recovery of eye positions from a population of complex gain fields in which each gain field was normalized to have the same mean value, averaging across all eye positions. Performance of normalized population was poor, despite the population still retaining a great diversity of gain field shapes reflecting the broad range of parameter values across the population. Physical eye positions were as shown in Figure 1.

Another way of inducing strong distortions in the recovered spatial map was to make all gain fields in the population have the same mean value. In general, each gain field will have a different mean value for its responses when averaged over all eye positions. However, the gain fields in a population can all be normalized to have the same mean value, even though the spatial pattern for each gain field remains different, and even though the population retains a broad range of values for all parameters. When that was done, the eccentricity of the spatial representation was completely disrupted. Comparing Figure 12A (un-normalized) and Figure 12B (normalized) shows an example of this, in which the normalized population of complex gain fields all have the same mean response, while retaining various orientations, spatial scales, translations, etc.

Note that Figure 12B (equalized mean responses for a population of complex gain fields) looks almost the same as Figure 11Bi (restricted translations for a population of planar gain fields). It happens that setting the translation parameter δ to be the same for all members in a population of planar gain fields has the effect of making all gain fields have the same mean value. On the other hand, setting the translation parameter to be the same for all members of elliptical paraboloid or hyperbolic paraboloid gain fields does not make them have the same mean value.

### AIT and LIP model gain field populations produced by a genetic algorithm

**Figure 13:**
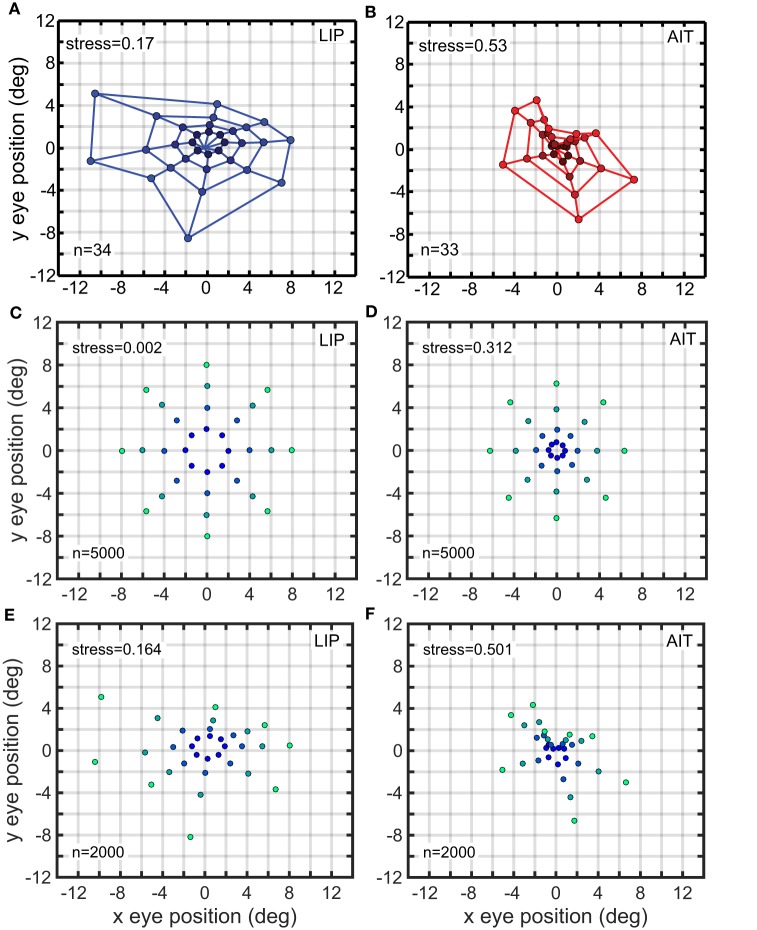
**Recovered eye-position spatial maps based on monkey data (A,B) or models of monkey data (C–F)**. **(A)** Recovered spatial map from data recorded in monkey lateral intraparietal cortex (LIP). **(B)** Recovered spatial map from data recorded in monkey anterior inferotemporal cortex (AIT). Compared to the LIP spatial map, the AIT map appears contracted toward the origin. **(C)** Recovered eye positions from a population of model complex gain fields whose parameters were selected using a genetic algorithm to produce an idealized version of the LIP monkey data in (**A**; veridical visual space). **(D)** Recovered eye positions from a population of model complex gain fields whose parameters were selected using a genetic algorithm to produce an idealized version of the AIT monkey data in (**B**; visual space contracted toward the origin). **(E)** Recovered eye positions from a population of model complex gain fields whose parameters were selected using a genetic algorithm to reproduce actual LIP monkey results in **(A)**. **(F)** Recovered eye positions from a population of model complex gain fields whose parameters were selected using a genetic algorithm to reproduce actual AIT monkey results in **(B)**. **(A,B)** Adapted from Sereno et al. (2014). Stress values are relative to physical eye positions.

We have previously computed eye-position spatial maps based on monkey data from LIP and AIT (Sereno et al., 2014). This was accomplished by applying identical MDS and Procrustes methods as described here to data from populations of neurons showing eye-position modulations in their responses. The spatial map for LIP corresponded to a fairly accurate representation of physical space (Figure 13A), keeping in mind the noisiness of the small population sample, while the spatial map for AIT was much more heavily distorted and appeared contracted toward the center (Figure 13B; compare with physical eye positions in Figure 1).

When applying the genetic algorithm to AIT and LIP data, we fit models to two versions of the data. One version was an idealization of the data, as described below. The other version was the original data shown in Figures 13A,B.

In the idealized version, the LIP spatial representation was portrayed as completely veridical, matching the physical eye positions (Figure 1), while the AIT spatial representation was contracted toward the central fixation point, with the contraction given by the function *r*′ = 0.143*r*^1.8^ where *r* is spatial eccentricity. These idealizations reflected our qualitative judgment of what constituted the essential features of the actual data. In creating the idealizations, we ignored orientational anisotropies as small-sample noise, as we don't currently have grounds to expect AIT and LIP to have different orientation properties. That may change in the future with additional data. The idealization focused on aspects of the data that appeared to account for much of the remaining variation and that was consistent with the general view that spatial response properties are different in dorsal and ventral visual streams in ways that may be functionally significant.

We consider the model fits to the idealized data first. We used a genetic algorithm to create a population of model complex gain fields (*n* = 500) that generated the idealized LIP eye-position spatial map, and also a second population of gain fields that generated the idealized AIT spatial map These spatial maps are shown in Figures 13C,D Then we compared parameter values for the gain field populations that generated the two spatial maps (AIT and LIP), looking for differences in the gain fields that could account for differences in the resulting spatial maps.

Each model gain field in the model populations was defined by 13 parameters, three for the sigmoidal component, and five each for the elliptical paraboloid and hyperbolic paraboloid components. Preliminary runs with the genetic algorithm identified six of those parameters as being important for differentiating idealized LIP and AIT spatial maps: the space constant parameter σ and the translation parameter δ for each of the three components (sigmoidal, elliptical, and hyperbolic) of complex gain fields, which were used in subsequent runs.

**Figure 14:**
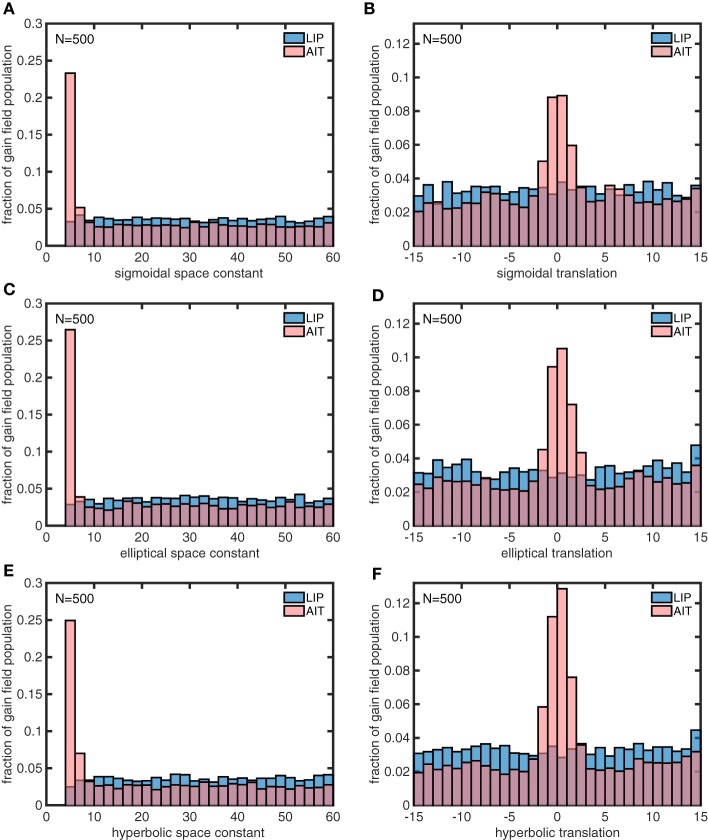
**Distributions of parameter values for model gain field populations that generated the spatial maps in Figures 13C,D, which are idealized versions of the monkey data (veridical for LIP, contracted toward the origin for AIT)**. The six panels show distributions for the following six parameters defining gain field shape for complex gain fields. **(A)** Sigmoidal space constant. **(B)** Sigmoidal translation constant. **(C)** Elliptical space constant. **(D)** Elliptical translation constant. **(E)** Hyperbolic space constant. **(F)** Hyperbolic translation constant. Histogram bars have been scaled so that their heights sum to 1.0, so bar heights indicate probabilities. These six parameters were found to be important for creating the difference between LIP and AIT eye-position spatial maps. AIT parameters are statistically biased toward smaller spatial space constant values, and smaller translation values (smaller dispersion), relative to LIP parameters. These histograms pool results from 10 runs of 500 gain fields each.

The model gain field parameters found using the genetic algorithm for the idealized AIT and LIP spatial maps (Figures 13C,D) are shown in Figure 14. The histograms in Figure 14 indicate that the distribution of the space constant parameter σ across the population was strongly skewed to smaller values in AIT compared to LIP (*p* < 10^−116^ under all conditions, using a rank sum test.). Essentially there was a subpopulation of gain fields in AIT that had very small σ values. Space constant values clustered around the lower bound enforced by the model, σ = 4, and even smaller values would likely have occurred if allowed. The translation parameter δ was also skewed to smaller values in AIT compared to LIP, producing a smaller gain field dispersion in AIT. Despite the σ and δ parameters being skewed to small values in AIT, the AIT distributions broadly overlapped with the LIP distributions over their entire ranges. There was no tendency for AIT and LIP parameters to clump into two disjoint regions of the parameter space.

**Figure 15:**
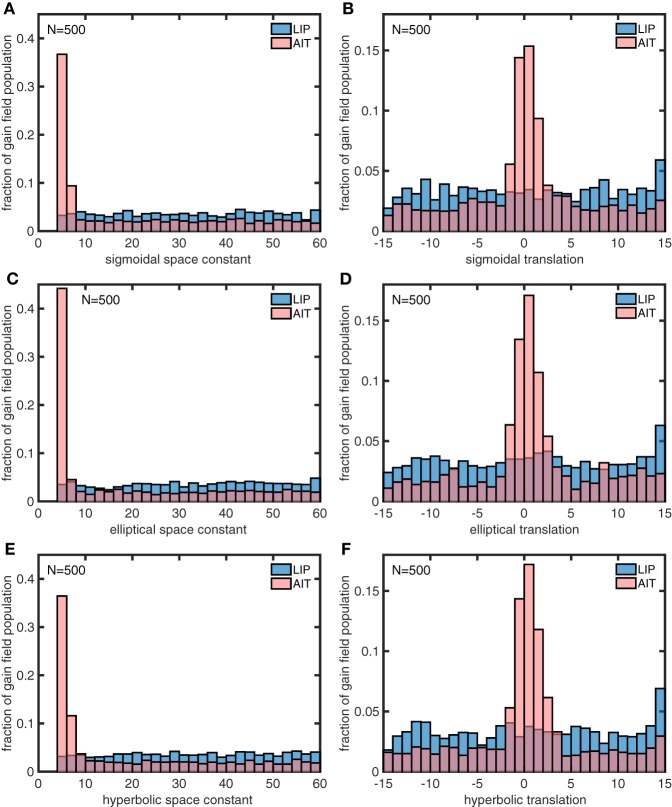
**Distributions of parameter values for model gain field populations that produced the spatial maps for LIP and AIT in Figures 13E,F, closely matching monkey data spatial maps (Figures 13A,B)**. The six panels show distributions for the following six parameters defining gain field shape for complex gain fields. **(A)** Sigmoidal space constant. **(B)** Sigmoidal translation constant. **(C)** Elliptical space constant. **(D)** Elliptical translation constant. **(E)** Hyperbolic space constant. **(F)** Hyperbolic translation constant. Parameter values were found using a genetic algorithm trained on the monkey data. These parameter values closely resemble parameter values producing spatial maps fitted to idealized monkey data (Figure 14). Again, AIT parameters are statistically biased toward smaller spatial space constant values and smaller translation values (smaller dispersion), relative to LIP parameters. The similarity between gain field parameters for idealized and actual spatial maps for LIP and AIT suggests that the idealizations capture essential differences between LIP and AIT spatial maps. These histograms pool results from 4 runs of 500 gain fields each.

In a similar manner, we also used the genetic algorithm to generate gain field parameters that produced spatial maps corresponding to actual LIP and AIT spatial maps (Figures 13E,F) rather than the idealized spatial maps (Figures 13C,D). The distributions of parameters for the actual maps (Figure 15) closely resemble the parameters for the idealized maps (Figure 14). This suggests that the idealized spatial maps (veridical for LIP, contracted for AIT) captured what we viewed as the essential differences between the actual LIP and AIT spatial maps.

## Discussion

We found that all gain field shapes tried, including both purely planar and those with no planar component (elliptical and hyperbolic paraboloids), were capable of performing an accurate recovery of eye-position space. This accurate recovery of space depended on a broad diversity of gain fields within the population. Recovery failed when gain field parameters were restricted to a narrow range (e.g., restricted orientations or space constants), with the nature of the failure depending on both the gain field category as well as the particular parameter being restricted. Finally, using a genetic algorithm to produce populations of gain fields that could mimic spatial maps recovered from monkey neurophysiological data, we found that AIT gain fields tended to be more foveally dominated than LIP gain fields, having smaller dispersions from the fixation point and smaller space constants.

Although the literature tends to emphasize gain fields as having a prominent planar aspect, in reality most gain fields have more complex shapes than that, as was outlined in the introductory section. Our finding that gain fields with no planar component (elliptical and hyperbolic paraboloids) work just as well as pure planes suggests that attempts to squeeze gain field data into the category of “planar” may be misplaced, although perhaps convenient for certain types of modeling. If a biological gain field happens to have a strong planar component, it is because the presence or absence of a planar component doesn't matter, not because a planar component is necessary for gain field function.

Moreover, from an evolutionary or developmental perspective, creating planar gain fields from nonlinear neural components may be a more difficult task than creating more random-like complex gain fields. If linearity is not required for gain field populations to function effectively, then biologically it would seem there would be little drive to create planar gain fields.

On the other hand, in contrast to the view that gain fields are planar is a theoretical perspective contending that gain fields cannot be purely planar but must have a nonlinearity of some sort in their shape (for example, distorting a plane into a sigmoid; Pouget and Sejnowski, 1997; Pouget and Snyder, 2000). For our purposes and methods, it made no significant difference if the gain field was linear or nonlinear. Linear (planar) and nonlinear (sigmoidal) gain fields worked essentially equally well in reconstructing eye-position spatial maps, as did gain fields with stronger nonlinearities than sigmoidal (Figures 7, 8). Our modeling suggests that while gain fields aren't required to be planar, there is no reason in principle why they couldn't be planar.

Characteristics of a gain field population defined the nature of the visual space derived from it. For example, restricting the range of gain field orientations, gain field spatial space constants, or gain field translations in the population disrupted spatial representations in different ways (Figures 10, 11). In a similar manner, one would expect that differences in gain field properties for different brain areas would be reflected in different characteristics for eye-position visual space in those brain areas.

Eye-position spatial maps are substantially different in LIP, a dorsal visual area, and AIT, a ventral visual area (Sereno et al., 2014). While the LIP spatial map (Figure 13A) is an approximately veridical representation of physical visual space (shown in Figure 1), the AIT map (Figure 13B) is more distorted, appearing contracted toward the center. While that previous work established that eye-position spatial maps differ in LIP and AIT, this study extends that by exploring the effects of different gain field shapes and parameters on spatial maps and using a genetic algorithm to determine how gain fields may differ in AIT and LIP to produce those different spatial maps.

Differences in eye-position spatial maps in different brain areas may reflect differences in processing goals for those areas. The spatial map in LIP may provide a coordinate representation of physical space in order to carry out accurate visuomotor control of movements within the physical world, such as grasping. The more distorted spatial map in AIT may provide a more abstract, categorical representation of space (i.e., to the left of, on top of, etc.), perhaps a useful representation for encoding relations of objects within scenes into memory, or part/whole relationships in the representations of objects. The coordinate/categorical terminology derives from studies by Kosslyn and colleagues (Kosslyn et al., 1989, 1992; Kosslyn, 2006). Eye position is just one spatial cue, and there may be many different concurrent spatial representations across cortical regions, dependent on the availability or input of different cues or combinations of cues.

In this study, we found it surprisingly easy to generate model gain field populations that yielded accurate representations of eye-position space, as occurs in LIP, simply by using gain field parameters that followed a uniform random distribution. In contrast, it was much harder to create the more distorted spatial map similar to that found in AIT, to the extent that it was not possible to do so by setting gain field parameters manually and we had to resort to using a genetic algorithm. This suggests that the distorted spatial map in AIT is not some random epiphenomenon in an area where spatial representation isn't important. Rather the distorted spatial map in the ventral stream likely reflects some specific spatial representation(s) with particular constraints perhaps driven by a particular function(s), which we don't fully understand yet.

We used a genetic algorithm to develop two populations of model complex gain fields that could generate spatial maps matching those derived from LIP and AIT data. The statistical distributions of parameters for the two gain field populations are dramatically different (Figure 15). These modeling results constitute a prediction of statistical differences between the shapes of LIP and AIT gain fields across their respective populations. Unfortunately, within the current data, gain fields have not been measured at high resolution (using a large number of gaze angles) and sampled over a large population, to enable empirically well-defined measures of gain field population characteristics. The demonstration that (1) gain field populations can be used to generate eye-position spatial maps, (2) eye-position spatial maps differ in different cortical areas, and (3) differences in spatial maps in different areas may be explained by differences in gain field characteristics, may serve as a motivation in future studies to better quantify properties of gain fields, as well as provide a guide as to which parameters may be most useful to measure.

Differences between spatial characteristics of AIT and LIP gain fields found here are analogous to previously reported differences between AIT and LIP receptive fields. We have previously studied retinotopic spatial maps in LIP and AIT based on receptive fields in those areas, both experimentally (Sereno and Lehky, 2011) and theoretically (Lehky and Sereno, 2011). Similar to what was found here for eye-position spatial maps, we found that the retinotopic spatial map for AIT neural populations was more distorted than the LIP retinotopic map. Further, we demonstrated with modeling that this difference could be explained if AIT receptive fields had smaller translations (smaller RF dispersion) and smaller spatial scale (smaller RF diameter) than LIP gain fields. Through modeling in this study, we provide here the first evidence that analogous differences between AIT and LIP gain fields (smaller dispersion and smaller spatial scale) can account for differences in the eye-position spatial maps for those areas. Thus, it appears that both receptive field and gain field populations are more foveally dominated in the ventral stream compared to the dorsal stream.

It is not surprising that the spatial scale of receptive fields or of gain fields is an important parameter determining the characteristics of spatial maps derived from those fields. What is counterintuitive here is that space is encoded more accurately with large fields than small ones. LIP encodes more accurate representations of eye-position space than AIT (Figure 13), corresponding to larger mean values for the space constants in the underlying gain fields of LIP (Figures 14, 15). Similarly, in previous modeling we found that larger values for scale in receptive fields (larger RF diameters) led to more accurate reconstruction of retinotopic space (Lehky and Sereno, 2011). This is opposite to the traditional systems neuroscience view that large diameter fields lose spatial information (i.e., are involved in producing positional invariance), and that small fields produce the most accurate representation of space (see e.g., Serre et al., 2007). Our perspective on the superiority of large fields for encoding space has been discussed in Lehky and Sereno (2011), Sereno and Lehky (2011), and Lehky et al. (2013).

Less obvious is another parameter we have identified as critical for determining spatial maps, namely the dispersion of the RF population or of the gain field population, which is a measure of translation rather than scale. Restricting the dispersion of gain fields (Figure 11B) severely distorts the eye-position spatial map, and analogously restricting the dispersion of RFs distorts retinotopic maps (Lehky and Sereno, 2011). While RF dispersions have been shown to differ in different cortical areas [for example AIT dispersion (Tovée et al., 1994; Op de Beeck and Vogels, 2000) is smaller than LIP dispersion (Ben Hamed et al., 2001)], we are not aware of any characterizations of gain field dispersions. Our modeling of the differences between LIP and AIT suggests that gain field dispersion in AIT will be more restricted than LIP.

A common interpretation for eye-position gain fields is that they are used to perform a coordinate transform from retinotopic coordinates to head-centered coordinates (Zipser and Andersen, 1988; Pouget and Snyder, 2000; Salinas and Abbott, 2001). A consequence of this idea is that getting to an allocentric visual reference frame requires multiple coordinate transforms, whether arranged in a series (retina-centered to head-centered, head-centered to body-centered, body-centered to world-centered) as suggested by Andersen et al. (1993), or multiplexed concurrently within a single network (Blohm et al., 2009; Blohm, 2012).

However, given that activity in a gain field population by itself can directly decode eye position in both dorsal and ventral streams independent of any retinotopic information, a different perspective from the traditional coordinate-transform approach can be developed. This may provide an alternative to the coordinate-transform approach under certain conditions but does not necessarily replace it. We suggest that an allocentric spatial map might be built up in primates over time by successively fixating different locations using a population of cells that are modulated by angle of gaze. That is consistent with our previous finding (Sereno et al., 2014) that a single spatial cue in itself, eye position, provides sufficient information to recover space. Recent psychophysical evidence is supportive of the existence of a memory store that over time builds up a representation of the current scene, or integrates parts of complex objects, during multiple fixations [see Zimmermann et al. (2014); also see Larochelle and Hinton (2010) for an example of neural modeling that integrates information over multiple fixations]. It should be noted that an intrinsic approach to decoding gain fields (Lehky et al., 2013), exemplified by this model, inherently represents positions in a relational manner (i.e., positions are encoded with respect to each other), which is the essence of an allocentric representation.

In conclusion, we have shown that accurate encoding of eye-position space can be carried out by populations of gain fields largely independently of the shapes of the gain fields. In particular, gain fields do not need to be planar or any other simple geometric shape. Further, we demonstrated that the characteristics of gain fields determine the geometry of eye-position space, so that different gain field properties in different brain areas will lead to different representations of space. We have predicted specific quantitative differences between gain fields for two brain areas, AIT and LIP, associated with different spatial maps. These findings identify and clarify characteristics of gain fields that influence spatial encoding. They provide insight into the functional consequences of having different eye-position modulations in different brain areas, and give motivation to experimentally quantify gain field properties more extensively. Overall, these findings give indications as to how gain field characteristics may influence behavior through the construction of our many senses of space.

## Author contributions

SL did the modeling. SL, AS, and MS wrote the manuscript.

### Conflict of interest statement

The authors declare that the research was conducted in the absence of any commercial or financial relationships that could be construed as a potential conflict of interest.
